# Eugenol modulates the NOD1-NF-κB signaling pathway *via* targeting NF-κB protein in triple-negative breast cancer cells

**DOI:** 10.3389/fendo.2023.1136067

**Published:** 2023-02-27

**Authors:** Xiaoyu Shi, Weiwei Zhang, Xiao Bao, Xiaozhu Liu, Ming Yang, Chengliang Yin

**Affiliations:** ^1^ Department of Pharmacy, Yantai University, Yantai, China; ^2^ Pharmacy Department, Wenzhou Nursing School, Wenzhou, China; ^3^ Department of Cardiology, The Second Affiliated Hospital of Chongqing Medical University, Chongqing, China; ^4^ Obstetrics Department, The First Dongguan Affiliated Hospital of Guangdong Medical University, Dongguan, China; ^5^ Macau University of Science and Technology, Faculty of Medicine, Macau, Macau SAR, China

**Keywords:** eugenol, triple-negative breast cancer, CMAP, proteomics, DARTS, target protein

## Abstract

**Background:**

The most aggressive subtype of breast cancer, triple-negative breast cancer (TNBC), has a worse prognosis and a higher probability of relapse since there is a narrow range of treatment options. Identifying and testing potential therapeutic targets for the treatment of TNBC is of high priority.

**Methods:**

Using a transcriptional signature of triple-negative breast cancer collected from Gene Expression Omnibus (GEO), CMap was utilized to reposition compounds for the treatment of TNBC. CCK8 and colony formation experiments were performed to detect the effect of the candidate drug on the proliferation of TNBC cells. Meanwhile, transwell and wound healing assay were implemented to detect cell metastasis change caused by the candidate drug. Moreover, the proteomic approach was presently ongoing to evaluate the underlying mechanism of the candidate drug in TNBC. Furthermore, drug affinity responsive target stability (DARTS) coupled with LC-MS/MS was carried out to explore the potential drug target candidate in TNBC cells.

**Results:**

We found that the most widely used medication, eugenol, reduced the growth and metastasis of TNBC cells. According to the underlying mechanism revealed by proteomics, eugenol could inhibit TNBC cell proliferation and metastasis *via* the NOD1-NF-κB signaling pathway. DARTS experiment further revealed that eugenol may bind to NF-κB in TNBC cells.

**Concludes:**

Our findings pointed out that eugenol was a potential candidate drug for the treatment of TNBC.

## Highlights

1. Eugenol inhibits the proliferation and metastasis of triple-negative breast cancer cells;
2. Eugenol plays an anti-tumor role by inhibiting the NOD1-NF-κB signal pathway;
3. Target protein of eugenol in triple-negative breast cancer cells is NF-κB.

## 1 Introduction

Triple-negative breast cancer (TNBC) is a subtype of breast tumor that makes up between 15% and 20% of all breast malignancies and is identified immunohistochemically by the absence of the estrogen receptor, progesterone receptor, and human epidermal growth factor receptor-2 (1, 2). Compared with other types, TNBC has a strong metastatic capacity, rapid proliferation, and poor prognosis (3). Due to these aggressive features and lack of specific molecular therapeutic targets, traditional chemotherapies remain the standard of care for TNBC patients (4). However, it has been reported that responses to chemotherapies are usually short and followed by rapid relapse, and common lung, and brain metastases (5), and TNBC patients who develop metastatic disease only have 13-18 months of median OS (6). Therefore, it is urgent to find new drugs to treat triple-negative breast cancer.

Connectivity Map (CMap, http://www.broad.mit.edu/CMap/) is a database, which contains 7000 microarray expression profiles from different cancer cells treated by 1309 molecular drugs (7). Researchers can quickly use gene expression profile data to compare drugs highly related to diseases and summarize the possible mechanism of action of drug molecules. Some outdated molecules have been successfully repositioned by comparing the transcriptome treated by compound, through publicly available resources like Gene Expression Omnibus, which is freely accessible (8). For example, Li et al. performed CMap analysis of the transcriptional signature obtained from GEO and identified aspirin has potential for combination therapy in cancers (7). In addition, Yin et al. also reported several candidate drugs for sclerosis treatment through drug repositioning by CMap. Based on this idea, we found that eugenol can change the expression of associated genes in TNBC cells *via* CMap.

Eugenol is a natural compound in clove oil and other spices such as basil, cinnamon, and bay leaves (9). Many works of literatures reported that eugenol has several physiological functions, such as antiseptic (10), analgesic, and anti-bacterial (11). Besides, eugenol also has some effects on the proliferation and metastasis of many tumors. For example, Eugenol’s ability to inhibit the growth of malignant melanoma cells and promote apoptosis was described by Marina Pisano (9). Li discovered that eugenol had tumor-suppressive properties in lung cancer (12). According to Cui, eugenol may eventually contribute to its anti-tumor activity by suppressing NF-κB expression in the NSCLC (13). Many studies have reported that eugenol inhibits breast cancer (14), cervical carcinoma (15), prostate cancer (16) and so on. However, the detailed mechanism of eugenol in TNBC, especially its target protein, has not been studied yet.

MS-based proteomics have progressed from simple protein sequencing to a powerful approach for identifying disease patterns and signatures, revealing molecular mechanisms of preventive drugs (12). Label-free quantitative proteomics can determine the abundance of the proteins across diverse samples, it has also been a promising method to explore the mechanism of action of drugs (17). Besides, target identification using drug affinity responsive target stability (DARTS) is consistently used to determine the molecular targets of small-molecule compounds (18). It is primarily based on the observation that proteins are less likely to undergo proteolysis in the presence of compound than they are in the absence of one, making it potentially useful for identifying any small-molecule target.

Our study aimed to find natural compounds to treat TNBC through CMap analysis. We used CMap to analyze the genomic changes of TNBC cells obtained by GEO, and carried out anti-cancer research on the top candidate natural compound—eugenol. The effect of eugenol on the proliferation and metastasis of TNBC was studied by CCK8, colony formation, wound healing and transwell experiments. Using label-free proteomics to reveal the mechanism of eugenol in TNBC. Importantly, DARTS experiment combined with MS detection revealed the possible target of eugenol in TNBC cells. The results showed that eugenol, as a natural compound, has significant anti-tumor effects on TNBC proliferation and metastasis, and probably played an anti-tumor role by influencing NOD1- NF-κB signal pathway. It was vital that we found that NF-κB may be the target protein of eugenol in TNBC cells.

## 2 Materials and methods

### 2.1 Cell culture and reagents

MDA-MB-231 and MDA-MB-453 cells were cultivated in 1640 medium (11875119, Gibco, Grand Island, USA) and Leibovitz’s L-15 medium, respectively (11415064, Gibco, Grand Island, USA). At 37°C in a humidified incubator with 5% CO2, all culture mediums were supplemented with 10% fetal bovine serum (04-001-1ACS, Biological Industries, Kibbutz Beit Haemek, Israel), 100 U/ml penicillin G (15070063, Thermo Fisher Scientific, Waltham, USA). The following sources provided the specific primary antibodies: anti-NOD1 (ab215726, Abcam, Cambridge, USA), anti-NF-κB (66535-1-Ig, Proteintech, Wuhan, China), anti-NF-κB (phospho S536) (ab76302, Abcam, Cambridge, USA), anti-IKB alpha (10268-1-AP, Proteintech, Wuhan, China), anti-phospho-IKB alpha (SAB5700432, Sigma, St. Louis, USA), and anti-GAPDH (60004-1-Ig, Proteintech, Wuhan, China).

### 2.2 Drug screening *via* the CMap

In this study, four GEO datasets (GSE38959, GSE45827, GSE65194, GSE61724) were used, and the cut-off criterion for up and down probe sets, which were used to query CMap, was a two-fold change (7).

### 2.3 Cell viability assay

Cells were seeded in 96-well plates at a density of 2000 cells per well, eugenol group was added after 24 h of culture, three replicate wells were set for each concentration and cultured separately. 10 μL of a CCK8 solution containing 5 mg/mL were added to each well and incubated for 1 h after the indicated times of 24 h, 48 h, and 72 h. After 1 h, discard the supernatant, use a microplate reader to detect the absorbance at 450 nm.

### 2.4 Colony formation assay

Six-well plates were used for 1 × 10^3^ cell seeding. The cells were combined, and they were then cultivated for 14 days in 10% FBS culture media. A single colony was defined as a cluster of 30 cells. Eugenol group was mixed in complete culture medium with different concentration eugenol. Then the cells were fixed, stained with crystal violet and counted.

### 2.5 Assays for cell migration and invasion *in vitro*

The migration & invasion was measured by transwell chambers coated with fibronectin (for migration) & Matrigel (for invasion) on polycarbonate filters, respectively. The cells (1×10^5^) were plated on the upper surface of a filter with eugenol plus medium without serum. The medium was added into the lower chamber whether there is or there is not 10% of FBS. Cells that migrated (7 h later) or invaded (20 h later) through the filter were stained with crystal violet and then counted after taking photo under microscope (Nikon, Japan).

### 2.6 Wound healing assay

The published method was used to evaluate the wound healing assay for cell migration. In a 6-well plate with finished media, cells proliferated until confluent. Using a 100 μL pipette tip, the wounds were scraped. Following a 24-hour treatment with varied Eugenol concentrations, the cell debris was washed with serum-free media. With the same field of view and a light microscope, the wounds were photographed at 0 and 48 hours, respectively (Nikon, Tokyo, Japan). A microscope was used to assess the relative distance between the scratches, and ImageJ was used to assess it.

### 2.7 Label-free proteomics

Collect the cells according to the routine operation. After ultrasonic crushing, centrifuge to extract the total protein of the cells. After protein quantification with BCA, 50 μg protein was taken from each group for DTT and IAA treatment, and was digested by trypsin overnight. After concentrating the sample, loading 2 μg sample for LC-MS analysis. Orbitrap Fusion (Thermo Fischer Scientific) was used for mass spectrometer (MS) analyses. Raw data is searched by MaxQuant search engines (Thermo Fischer Scientific), and subsequently corrected by Persus software. Foldchange mean eugenol treatment/DMSO treatment. Log2Foldchange ≥ 0.6-fold or ≤-0.6-fold cut-off value was used to identify up-regulated and downregulated proteins with a p value < 0.05.

### 2.8 Drug affinity responsive target stability

The DARTS experiment was conducted as following (18). After cells were washed and then lysed with protein extraction kit (Beyotime, Haimen, China) including a protease inhibitor cocktail (Sigma-Aldrich). After centrifugation (14,500 rpm for 15 min, the lysates were mixed with 10×TNC buffer [0.1M CaCl2, 0.5M NaCl, 0.5M Tris·HCl (pH 8.0). Then, the lysates were incubated with DMSO (0.05%) and eugenol, respectively for 35 min at room temperature after incubated 60 min at 4°C. After the treatment, the samples were proteolyzed respectively in 0 and 0.1% (1:1000) of pronase for 15 min at room temperature. The samples were added to 2 liters of ice-cold buffer containing a 20-protease inhibitor cocktail to halt proteolysis, and they were then put on ice right away. The samples were then boiled for 5 minutes at 100^°^C while being combined with 5 samples of loading dye. The equal amount of each treated samples was then added into gels of SDS‐PAGE for Coomassie Blue staining or Western blot after running the gels.

### 2.9 Transfection of NF-κB p65 short RNA

Briefly, NF-κB p65 shRNA plasmid (Santa Cruz Technology Inc., Shanghai, China) was transfected into MDA-MB-231 cells using Lipofectamine in accordance with the manufacturer’s instructions. The negative control was ncNF-κB p65 scrambled non-targeting ncRNA. After 48 h, transfected cells were used for the CCK8 assay for the growth and transwell chamber assay for migration, as well as Western blot analysis for protein expressions mentioned-above.

### 2.10 Western blot analysis

Western blot experiment was carried out according to conventional methods (19). Proteins from the entire cell were isolated, separated on SDS-PAGE gels, and then transferred to PVDF membranes. 5% nonfat dried milk was used to block membranes for 1 hour at RT, then incubation with the primary antibodies (1:500) overnight. After washing, using secondary antibodies (1:1000) to incubate the membranes at RT for 1 h. The ECL detection system (Santa Cruz, USA) was utilized to visualize immunoreactive protein bands.

### 2.11 Statistical analysis

The data from more than two times of independent experiments were analyzed using SPSS 16.0 software. Data were expressed as mean ± SD and analyzed using the student’s t-test, and a P value of 0.05 was regarded as statistically significant.

## 3 Results

### 3.1 Using CMap to find drugs for triple negative breast cancers using gene signatures

**Figure 1:**
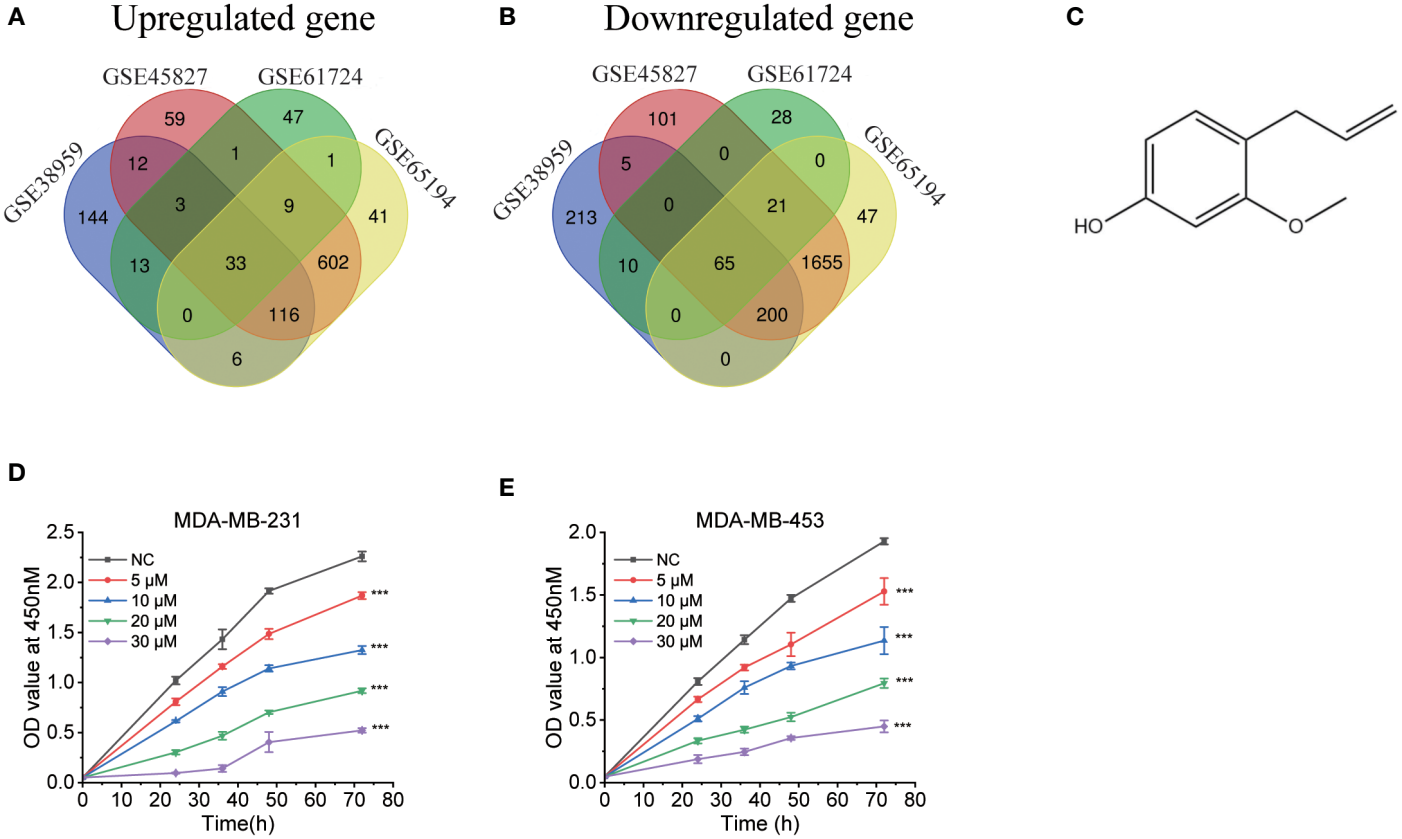
Bioinformatics analysis to explore the anti-tumor potential of eugenol. **(A)** The Venn diagram displays the information of distinct genes that are increased across four data sets (GSE45827, GSE38959, GSE61724, GSE65194) to query the CMap. **(B)** The Venn diagram displays the amount of downregulated different genes vis four data sets (GSE45827, GSE38959, GSE61724, GSE65194) to query the CMap. **(C)** The chemical structure of eugenol. **(D, E)** CCK8 assay of indicated MDA-MB-231 and MDA-MB-453 cells viability treated with eugenol (0-30 μM). Student’s t-test, n=3. ***P < 0.001.

We searched the GEO database for triple-negative breast cancers vs. respective normal tissue, then we obtained four datasets. **Table 1** included a summary of the data sources. All datasets had previously been published (20–23). Then, CMap was used to search for differentially expressed genes from each dataset. **Figures 1A, B** depict the intersection of normal vs. cancer datasets yielding 33 upregulated different genes and 65 downregulated different genes. We analyzed these 98 differential genes through CMap software, and the top 10 compounds were listed in **Table 2**. Other compound data were supplemented in **Table S1**. Interestingly, eugenol was the first natural product among the top 10 compounds. The chemical structure of eugenol was shown in **Figure 1C**. CCK8 experiment was used to detect the proliferation ability of eugenol of different concentrations on TNBC cells. The results showed that eugenol dose-dependently repressed the cell growth, which the IC50 values of growth repression were 16.84 and 15.81 µM for MDA-MB-231 cells and 27.25 and 16.99 µM for MDA-MB-453 cells while treatment for 48 h and 72 h (**Figures 1D, E**), respectively.

**Table 1:** A summary of the four datasets utilized for the CMap study (normal vs. tumor).

GEO	GSE45827	GSE61724	GSE38959	GSE65194
Pubmed ID	200045827	200061724	200038959	200065194
Normal	11	4	13	11
Tumor	41	16	30	14

**Table 2:** Top 10 list of drugs name after CMap analysis.

Rank	Score	ID	Name	Description
1	-99.4	BRD-K54256913	MK-1775	WEE1 kinase inhibitor
2	-99.08	BRD-K80527266	triacsin-c	Adrenergic receptor antagonist
3	-98.95	BRD-K32536677	AGK-2	SIRT inhibitor
4	-98.89	BRD-K01192156	tyrphostin-AG-112	Protein tyrosine kinase inhibitor
5	-98.8	BRD-K86465814	HO-013	PPAR receptor agonist
6	-98.73	BRD-K32977963	eugenol	Androgen receptor antagonist
7	-98.66	BRD-K41260949	valproic-acid	HDAC inhibitor
8	-98.32	BRD-K82746043	navitoclax	BCL inhibitor
9	-97.99	BRD-K64451768	GANT-58	GLI antagonist
10	-97.92	BRD-U88459701	atorvastatin	HMGCR inhibitor

### 3.2 Eugenol displayed inhibition proliferation and metastasis in TNBC cells

**Figure 2:**
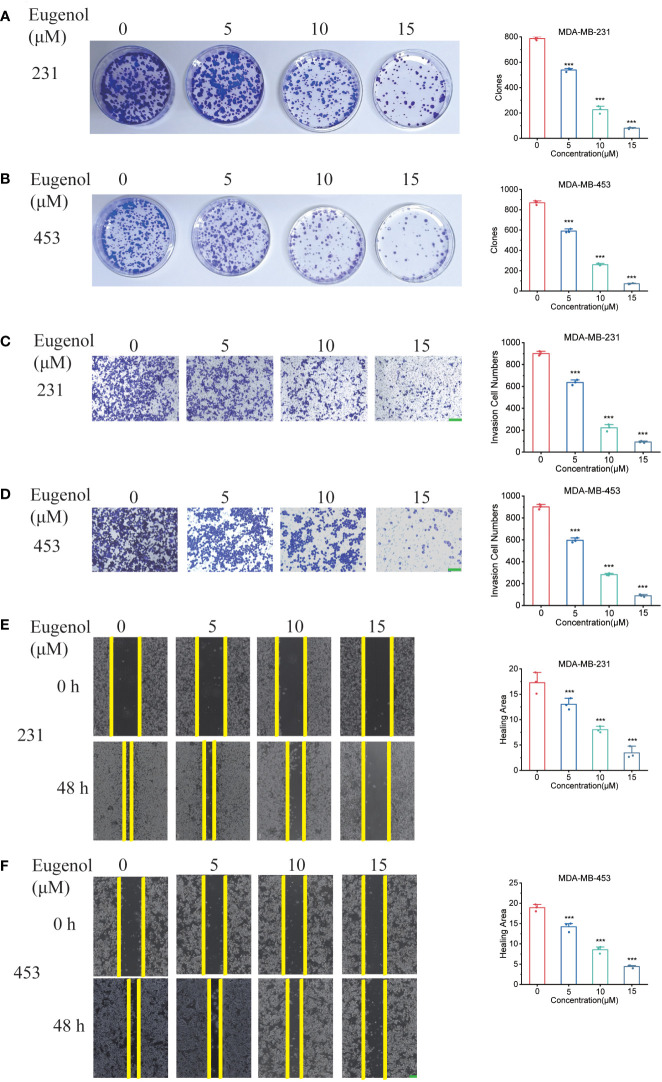
Eugenol inhibits TNBC cell metastasis and proliferation. **(A, B)** Colony formation assay for proliferation ability in MDA-MB-231 and MDA-MB-453 cells after eugenol treatment. **(C, D)** Detecting the ability of cell metastasis after eugenol treatment by transwell assay in MDA-MB-231 and MDA-MB-453 cells. Scale bar:green, 200μm. **(E, F)** Wound healing analysis of migration of MDA-MB-231 and MDA-MB-453 cells treated with eugenol for 48 h. Scale bar:green, 200μm. The data are presented as the means ± SD. Student’s t-test, n=3. ***P < 0.001.

The results of colony formation assay also showed that eugenol inhibited the growth of TNBC cells *in vitro* in a dose-dependent manner (**Figures 2A, B**). Next, we tested and confirmed the suppressive activity of eugenol against the MDA-MB-231 (**Figure 2C**) & MDA-MB-453 (**Figure 2D**) cell migration and invasion. Eugenol dose-dependently suppressed the invasion and the relative ratios of cell invasion were decreased by 75.38% in MDA-MB-231 cells, and by 68.62% in MDA-MB-453 cells, respectively after treatment with eugenol at 15 µM. Corresponding, wound healing assay showed that the relative ratios of cell migration decreased by 64.2% in MDA-MB-231 cells, and by 68.51% in MDA-MB-453 cells, respectively after treatment with eugenol at 15 µM (**Figures 2E, F**). These results elaborated eugenol has shown the identical inhibitory trends of growth, migration in both cell lines.

### 3.3 Proteomics revealed the antitumor mechanism of eugenol

**Figure 3:**
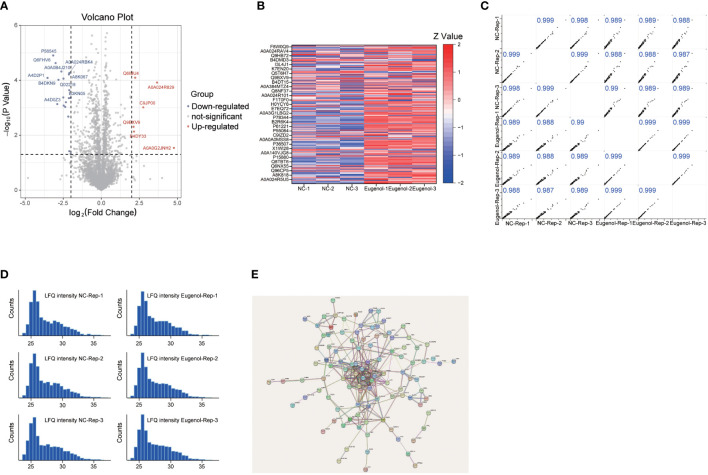
Lable-free proteomics reveals the anti-tumor mechanism of eugenol. **(A)** Volcano plot of identified proteins. The blue dot represented the down-regulated protein, the red dot represented the up-regulated protein, and the gray dot represented the protein with no significant difference. Student’s t-test (P value ≤ 0.05); **(B)** Heatmap of the differentially expressed proteins in two groups; **(C)** Pearson correlation showed clustering of samples replicate reproducibility; **(D)** The distribution of peptide length. **(E)** STRING PPI network analysis of the differentially changed proteins.

In order to further study the anti-tumor mechanism of eugenol, we conducted a label free proteomics experiment. First, MDA-MB-231 cells were treated with eugenol for 24h, while cells in the control group were treated with DMSO, then collected the cells and lysed them with NP40 lysate containing protease inhibitor. After BCA quantification, take 100 μg protein for sample loading pretreatment and 12 h digestion by trypsin. Then, take 2 μg of loading samples from eugenol treatment group and DMSO group respectively, and using high-resolution liquid chromatography-mass spectrometry (LC-MS) for detection. The raw data were searched by MaxQuant and corrected by Persus software. We identified 3081 proteins in total, and the LFQ values of these two groups were analyzed. Foldchange (FC= eugenol treatment/DMSO treatment) and P value were used to screen significantly different proteins. We found that the two tested samples shared 3080 proteins in total (**Table S2**). The proteins with a log2fold change of 0.6 or -0.6 (eugenol vs. ctr) and a p-value of 0.05 were determined to have significantly increased and decreased in eugenol vs. control studies, respectively (**Figure 3A**). This resulted in 53 significantly up-regulated proteins (1.7% of the total) and 135 less up-regulated proteins (4.4% of the total). The expression of these 188 differential proteins is shown in the heatmap (**Figure 3B**). Then, to evaluate our dataset’s quality, we evaluated the correlation between the two groups of samples (**Figure 3C**). The results showed that R >0.9, and the histogram also showed that the sample protein shown normal distribution (**Figure 3D**). PCA analysis results also show that the two groups of data are separated significantly (**Figure S1**). The network was constructed as shown in **Figure 3E**. The most-connected proteins belonged to the family of RPL, including RPL3, RPL4, RPL7, RPL7A, RPL10, RPL10A, RPL15, RPL18A, RPL30 and RPS2.

### 3.4 Eugenol inhibits the proliferation and metastasis of TNBC cells by modulating NOD1-NF-κB signaling

**Figure 4:**
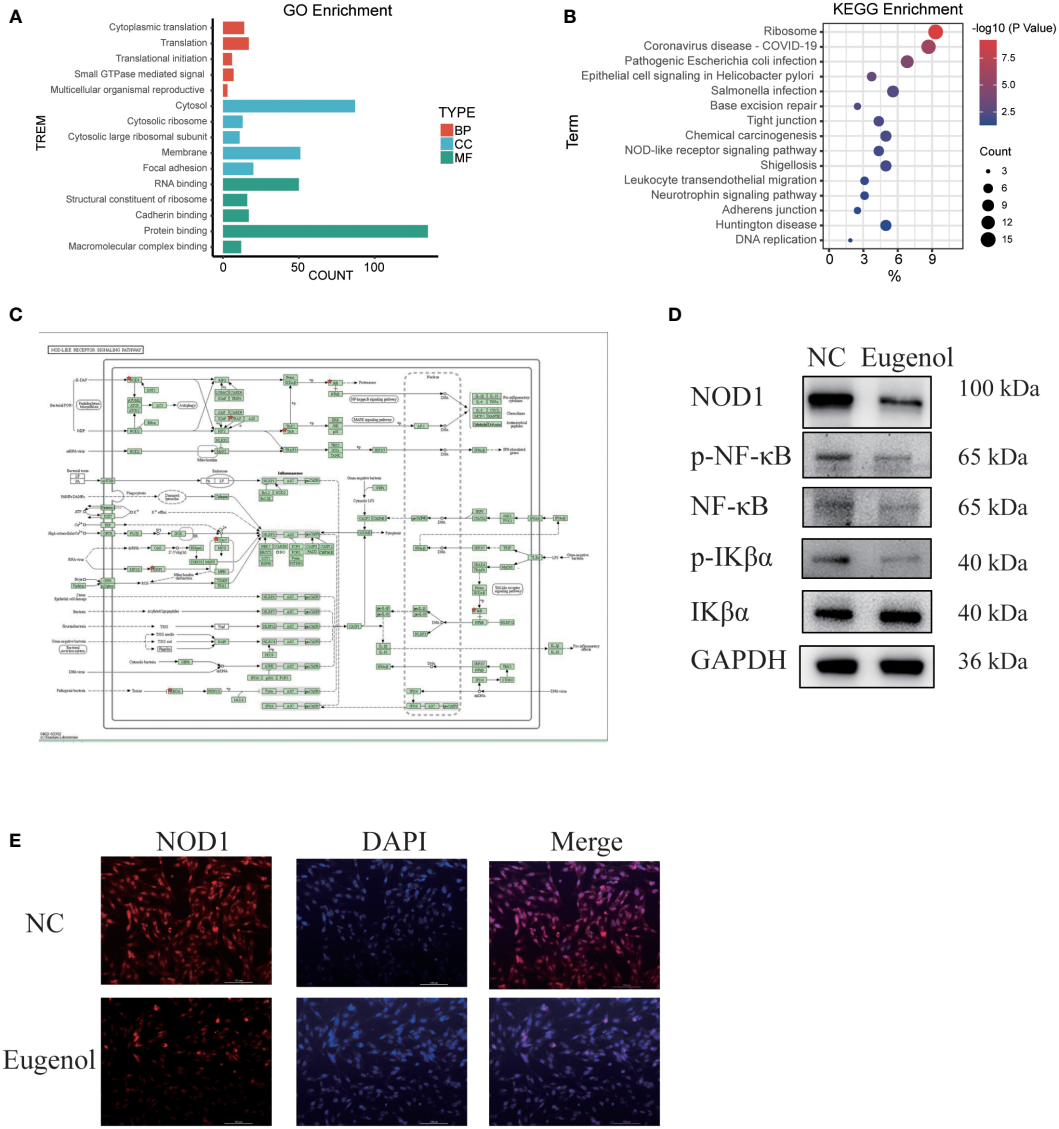
Main functional classification with the most significant enrichment of the differentially expressed proteins. **(A)** GO enrichment analysis of differentially abundant proteins in biological process, cellular component, molecular function. **(B, C)** KEGG pathway enrichment analysis of differentially abundant proteins. **(D)** Western blot experiment verified the effect of eugenol on signal pathway. **(E)** Detection of NOD1 expression after eugenol treatment by immunofluorescence assay.

In order to explore the action mechanism of eugenol, we used the DAVID website for Gene Ontology (GO) and Kyoto Encyclopedia of Genes and Genomes (KEGG) enrichment analysis of the above 188 differential proteins. Compared to control group, the different abundant proteins participated in 76 biological processes, which were manually grouped into 51 cellular components and 29 molecular functions. Biological process analysis indicated that these differential proteins were mainly related to cytoplasmic translation, translation, translational initiation, small GTPase mediated signal transduction and multicellular organismal reproductive process. Cellular component analysis revealed that most of the differentially abundant proteins belonged to the cytosolic ribosome, cytosol, cytosolic large ribosomal subunit, membrane and focal adhesion. Molecular function analysis revealed that most differentially abundant proteins were related to RNA binding, structural constituent of ribosome, cadherin binding, protein binding and translation regulator activity (**Figure 4A**). In addition, Eugenol strongly impacted the NOD-like receptor signaling pathway proteins, according to KEGG enrichment data (**Figure 4B**). As shown in **Figure 4C**, the proteins that affect the NOD-like receptor signaling pathway including NOD1, TRAF2, VDAC3, cDNA FLJ57740, DNM1L, TRIP9, TAB1, MAD3. To verify this result, we conducted western blot experiment, finding that eugenol can significantly reduce the expression of NOD1 and the phosphorylation expression of IKBα and NF-κB (**Figure 4D**). Moreover, the experimental results of immunofluorescence also showed that the expression of NOD1 protein decreased after eugenol treatment (**Figure 4E**). To sum up, the results demonstrated that eugenol can play an anti-tumor role by inhibiting the activation of NOD1-NF-κB pathway.

### 3.5 Drug affinity responsive target stability for eugenol target identification

**Figure 5:**
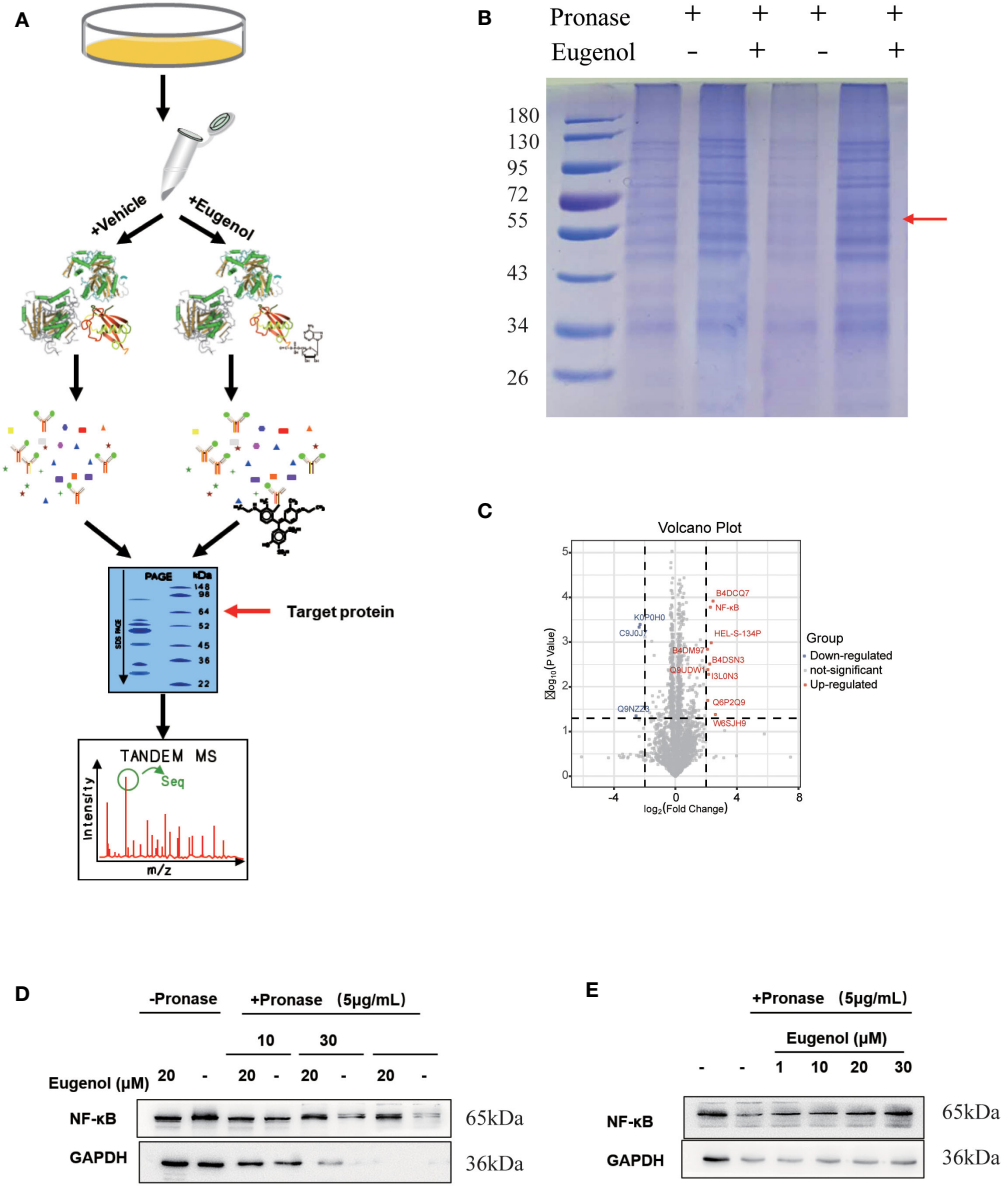
The target protein of eugenol in TNBC cells. **(A)** Work flow of drug affinity responsive target stability experiment; **(B)** MDA-MB-231 cells were treated with eugenol (20 μM), and lysates were subject to thermolysin digestion and Coomassie Brilliant Blue staining; **(C)** Enrichment of proteins in the protected band revealed by mass spectrometry analysis; **(D)** The DARTS assay for target validation. NF-κB protein stability was increased upon eugenol (20 μM) treatment in MDA-MB-231 lysates. Pronase treatment was conducted for 10, 30, and 60 min; **(E)** The DARTS assay demonstrated the dose-dependent binding of eugenol to NF-κB. Treatment with pronase (5 μg/mL) performed for 30 min “-” represent without the interference of pronase. “+” represent the interference of pronase.

Furthermore, a modified DARTS method was used to identify potential binding proteins of eugenol. This method is developed based on the observation of a molecule-protein complex. This complex may lead to their conformational changes, which could decrease in the sensitivity of enzyme digesting. MDA-MB-231 cells treated respectively with vehicle (0.05% of DMSO as the control) and eugenol in the absence or presence of 0.1% of pronase, were used for the DARTS experiment (**Figure 5A**). The protein band at 55-72KD on the SDS-PAGE gel for Coomassie Blue staining displayed the higher protein density in the treatment with eugenol +pronase (lane 2 and lane 4) compared to the treatment with DMSO + pronase (lane 1 and lane3) (**Figure 5B**). We collected protein samples with and without eugenol treatment, with three biological replicates in each group. Through the analysis of data by Pearson and PCA, it was found that the data presented high repeatability and separation (**Figures S2**-**S4**). Label-free proteomics technology was used for detection, and Persus software was used for data correction. The volcanic map displayed that the expression of NF-κB protein was significantly increased in eugenol +pronase compared to the treatment with DMSO + pronase (**Figure 5C**; **Table S3**). After treating with eugenol, the DARTS assay with immunoblotting revealed higher NF-κB stability against pronase, whereas the treatment with DMSO + pronase had no effect on proteolytic susceptibility (**Figure 5D**). Particularly, eugenol administration improved NF-κB stability depending on the dose, showing that eugenol selectively binds to NF-κB (**Figure 5E**). We have confirmed these results that NF-κB is recognized as a binding protein of eugenol in MDA-MB-231 cells.

### 3.6 Eugenol inhibits TNBC cell proliferation and metastasis by targeting NF-κB protein

**Figure 6:**
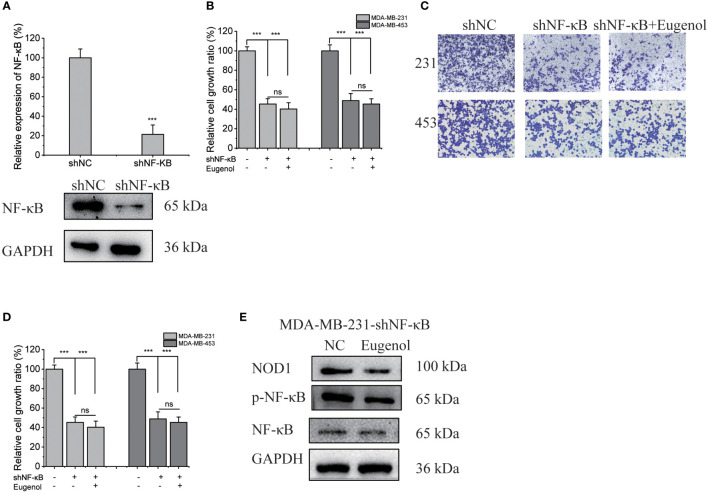
Verification of NF-κB as the target protein of eugenol in MDA-MB-231 cells. **(A)** Western blot experiment to verify the efficiency of transfection of shNF-κB. **(B)** CCK8 experiment to explore the proliferation ability of eugenol on NF-κB knockdown MDA-MB-231 and MDA-MB-453 cells. **(C, D)** Transwell experiment to explore the metastatic ability of eugenol on NF-κB knockdown MDA-MB-231 and MDA-MB-453 cells. **(E)** The expression of NOD1 in NF-κB knockdown MDA-MB-231 cells with or without eugenol treatment. Data presented as mean ± SD, n = 3. ^***^P < 0.001.

We next investigated whether the modulation of NOD1-NF-κB signaling by eugenol was initiated by targeting NF-κB. We used shNF-κB plasmid to transfect MDA-MB-231 cells, and obtained 231-KD cells with knockdown NF-κB expression. The transfection efficiency was verified by western blot assay and fluorescence microscope (**Figure 6A**, **Figure S5**). Meanwhile, 231-KD cells were used for cell function experiment, showing that the addition of eugenol had no significant effect on the proliferation of cells (**Figure 6B**). The findings demonstrated that eugenol had no effect on the growth of NF-κB knockdown cells. Compared with DMSO group, there was no significant change in cell invasion ability after adding eugenol (**Figures 6C, D**). Similarly, the results of wound healing experiment were the same (**Figures S6**-**S7**). Importantly, the eugenol therapy group compared to the control group in MDA-MB-231 NF-κB knockdown cells, western blot revealed that NOD1 expression was not significantly reduced (**Figure 6E**). To sum up, we found that eugenol may play its anti-tumor role by binding to NF-κB protein and inhibiting NOD1-NF-κB signaling pathway.

## 4 Discussion

Due to the special case characteristics of TNBC, there is a lack of targeted drugs, leading to chemotherapy becoming a conventional treatment method (24). However, the side effects of chemotherapy drugs which limit their clinical application (25). Therefore, new drugs and new therapies are greatly needed to treat triple-negative breast cancer. Because the development of new drugs is a time-consuming and expensive process, it appears that an approach is being made to reposition or repurpose known drugs to new indications, and some successful examples have been presented in some literature. Our study aims to reposition medications for the treatment of TNBC. We used the GEO database to look for tumor-related gene features (tumor and normal), then we used CMap data mining to look for prospective medications. Among them, we discovered that eugenol exerted an effective anti-tumor effect in TNBC cells, inhibiting the proliferation and metastasis of TNBC cells

CMap was developed to find drugs that caused gene expression patterns similar to or different from the disease of interest (26). As a result, it can be used to find new uses for “old” medications (7). GEO data is one of the greatest databases for gene expression information. We chose the TNBC data sets that included normal and tumor gene expression data. Then, we used the specified gene features derived from each data set to query CMap. There are 98 overlapping differentially expressed genes in 4 normal and malignant data sets. Thus, we discovered candidate pharmaceuticals among these medications that may play a role in the treatment of TNBC. Among the top 10 candidate compounds, we selected to focus on natural compounds. In fact, our study’s findings demonstrated that eugenol was a commonly utilized and secure medication that can be used to treat TNBC. Eugenol, according to our findings, prevented triple-negative breast cancer cells from proliferation and metastasis. This is consistent with the previous report by Abdullah, showing eugenol’s potential mechanism of action and anticancer effect (27). However, the target proteins of eugenol have not been studied in this literature.

Furthermore, the development of proteomics technology based on mass spectrometry can deeply reveal many diseases’ development and occurrence, and the process of drug action. Therefore, we used proteomic methods to explore the protein changes before and after eugenol treatment. This method can be more effective, fast, and accurate to study the mechanism of eugenol from a global perspective. Through this method, we found that eugenol can significantly inhibit the changes of proteins in the NOD1-NF-κB pathway, which is consistent with the previous research results.

An emerging pathogenic component in a variety of human cancers was dysfunctional NF-κB signaling. The anti-tumor potentials of targeting the NF-κB pathway have thus far been the subject of numerous experimental research and therapeutic exploitations (28, 29). According to this theory, our data showed that eugenol administration decreased the expression of the NOD1-NF-κB pathway, which ultimately aided in the drug’s tumor-suppressing effects. Consistent with our results, several studies have reported similar observations in different scenarios. For example, the study performed by Cui et al. (13) compared the relative expressions of NF-κB p65 in response to eugenol treatment and concluded that Eugenol repressed expression of NF-κB in NSCLS. Syed S Islam reported that eugenol enhanced cisplatin’s anti-cancer efficacy through inhibited the activation of NF-κB signaling in TNBC cells (30). All of these studies, including this research, suggested that eugenol may exert potent therapeutic effects against a number of human disorders by directly suppressing the NF-κB pathway.

The previous study conducted by Ibtehaj Al-Sharif et al. (31) for the first time disclosed the tumor suppressive role of eugenol in TNBC cells and attributed the eugenol-treatment had a strong effect on the expression of NF-κB. However, the clinical significance of this study is limited by the fact that it does not conduct a high-throughput full protein search, but focuses on the changes of a certain key protein, and cannot be explored globally. In this regard, here we revisited this important question with the employment of label-free proteomics to explore the possible mechanism of eugenol. Our data demonstrated that compared with the changes in whole protein after eugenol, the NOD1-NF-κB pathway were particularly significant changed. Although it is not the first report on eugenol’s anti-tumor effect on TNBC, this is the first report to reveal the anti-tumor mechanism and potential target proteins of eugenol in TNBC.

Noteworthily, the target protein of eugenol binding in TNBC cells has not been clarified. In this study, a modified “assay for drug affinity responsive target stability “DARTS” method and LC-MS technology were used to identify the potential binding proteins of eugenol (32). According to our knowledge, this was the first study to look into the potential binding protein of eugenol in TNBC cells. This developed approach is based on the observation that a compound interacts with a protein to form a compound-protein complex. Its complex could lead to conformational changes, which could reduce the sensitivity to enzyme digestion. MDA-MB-231 cells treated respectively with vehicle (0.05% of DMSO as the control) and eugenol in the absence or presence of 0.1% of pronase were used for the DARTS experiment. The DARTS assay coupled with the western blot analysis with NF-κB p65 antibody also exhibited a higher protein level in the treatment with eugenol + pronase compared to the treatment with DMSO + pronase. These results together have confirmed that NF-κB p65 is recognized as a binding protein of eugenol in TNBC cells. Finally, functional experiments were conducted after silencing NF-κB. As expected, the anti-tumor effect of eugenol was significantly reduced. This also proves that the potential target of eugenol in TNBC is NF-κB. This result provides detailed evidence for the application of eugenol in the treatment of triple-negative breast cancer, and provides the theoretical basis for eugenol in the treatment of triple-negative breast cancer.

## 5 Conclusions

In summary, we characterized eugenol administration significantly inhibiting the proliferation and metastasis of triple-negative breast cancer cells, mechanistically, our data highlighted eugenol could play an anti-tumor role by inhibiting NOD1-NF-κB signal pathway. Importantly, we identified NF-κB as a target protein of eugenol, which was subjected to the suppressive action of eugenol.

## Data availability statement

The data presented in the study are deposited in https://www.ebi.ac.uk/pride/archive/projects/PXD039305/private, accession number PXD039305.

## Author contributions

XS designed and wrote the manuscript. WZ and XB conducted statistical analysis. XL and MY drew graph. CY supervised the project. All authors performed the experiments, edited the manuscript and gave their final approval. All authors read and agreed to the final text.
