# Supplementary material for: Eugenol modulates the NOD1-NF-κB signaling pathway via targeting NF-κB protein in triple-negative breast cancer cells
Source: Front Endocrinol (Lausanne). 2023 Feb 27;14:1136067. doi: 10.3389/fendo.2023.1136067 (PMC10009163; doi:10.3389/fendo.2023.1136067)
Supplement: Supplementary file 1 [file DataSheet_1.docx]

Supplementary Material

Eugenol modulates the NOD1- NF-κB signaling pathway via targeting NF-κB protein in triple breast cancer cells

**Xiaoyu Shi1, Weiwei Zhang1, Xiao Bao2,** **Chengliang Yin3***

*** Correspondence:** Chengliang Yin Email: chengliangyin@163.com

# Supplementary Data

Supplementary Materials: Figure S1: PCA analysis diagram of eugenol treatment group and control group, Figure S2: Pearson correlation showed clustering of samples replicate reproducibility in DARTs experiment, Figure S3: The distribution of peptide length in DARTs experiment, Figure S4: PCA analysis diagram of eugenol treatment group and control group in DARTs experiment, Figure S5: Cell fluorescence diagram after transfection of NF-κB plasmid for 24h, Figure S6: Effect of eugenol on the metastasis of knockdown NF-κB MDA-MB-453 cells, Figure S7: Effect of eugenol on the metastasis of knockdown NF-κB MDA-MB-231 cells, Table S1: All compound data obtained by CMAP analysis, Table S2: Full proteome data obtained by label free analysis, Table S3: Full proteome data from Darts combined with LC-MS experiment.

# Supplementary Figures and Tables

## Supplementary Figures


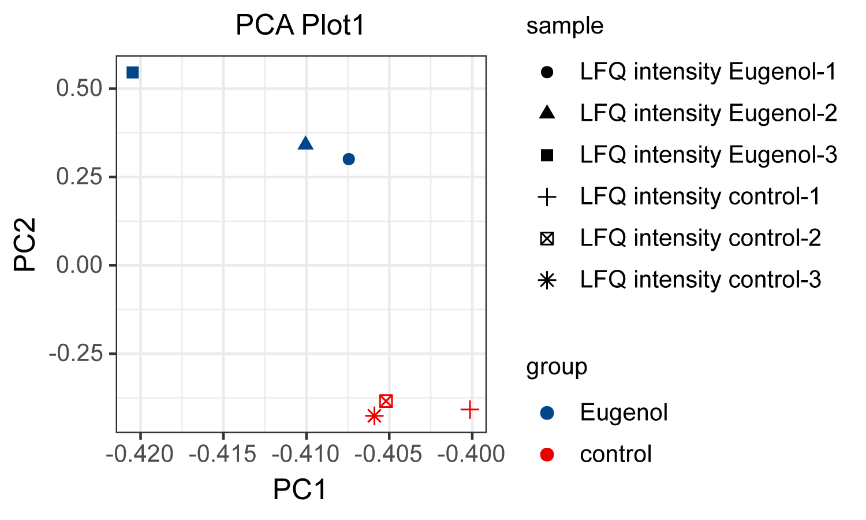


**Supplementary Figure 1.** PCA analysis diagram of eugenol treatment group and control group in label-free experiment.


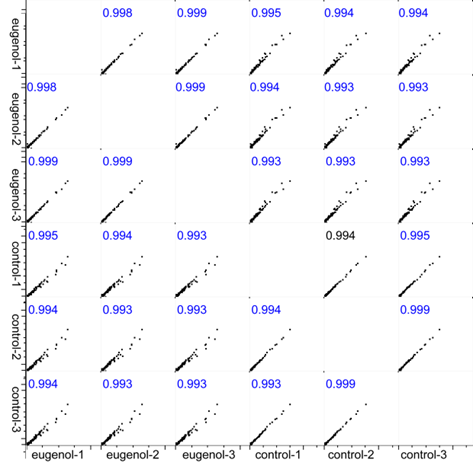


**Supplementary Figure 2.** Pearson correlation showed clustering of samples replicate reproducibility in DARTs experiment.


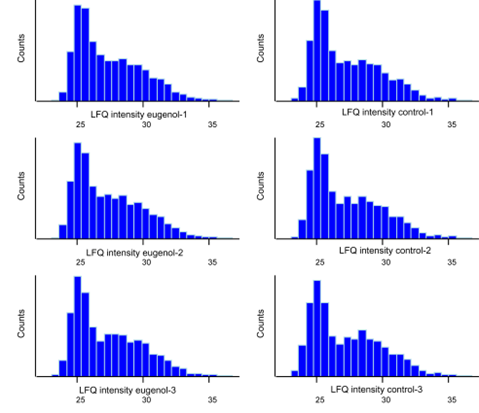


**Supplementary Figure 3.** The distribution of peptide length in DARTs experiment.


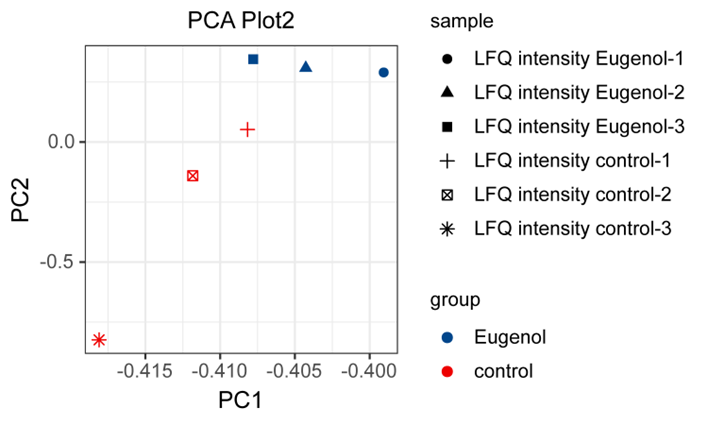


**Supplementary Figure 4.** PCA analysis diagram of eugenol treatment group and control group in DARTs experiment.


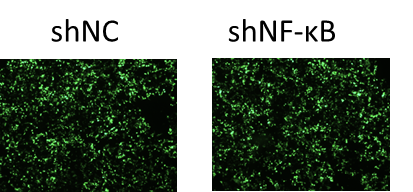


**Supplementary Figure 5.**  Cell fluorescence diagram after transfection of NF-κB plasmid for 24h.


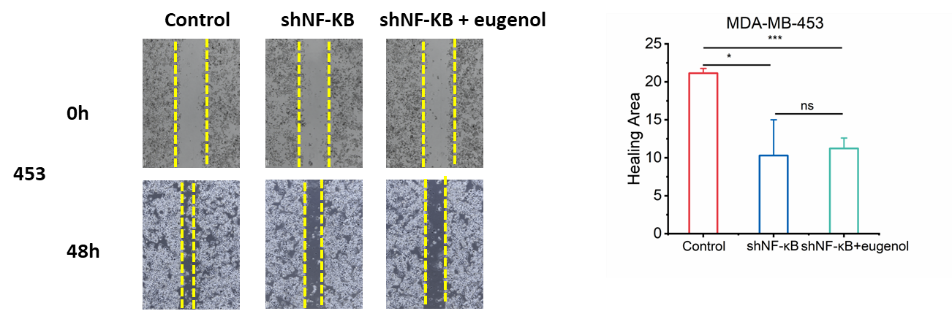


**Supplementary Figure 6.** Effect of eugenol on the metastasis of knockdown NF-κB MDA-MB-453 cells. The data are presented as the means ± SD. Student's t-test, n=3. *P < 0.05; **P < 0.01; ***P < 0.001.


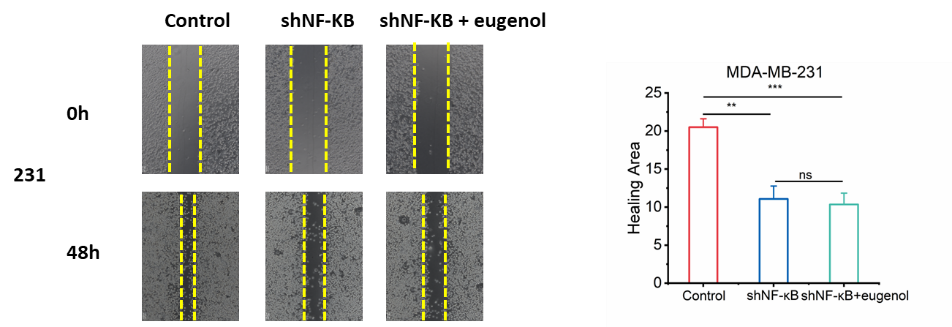


**Supplementary Figure 7.** Effect of eugenol on the metastasis of knockdown NF-κB MDA-MB-231 cells. The data are presented as the means ± SD. Student's t-test, n=3. *P < 0.05; **P < 0.01; ***P < 0.001.

## Supplementary Tables

**Supplementary Table S1:** All compound data obtained by CMAP analysis.

**Supplementary Table S2:** Full proteome data obtained by label free analysis.

**Supplementary Table S3:** Full proteome data from Darts combined with LC-MS experiment.
